# Cryptococcal Meningitis in an Apparent Immunocompetent Host

**DOI:** 10.7759/cureus.43121

**Published:** 2023-08-08

**Authors:** Guarina Molina, Maria A Perozo, Ruben Mora, John Stratidis, Nathalie Freites

**Affiliations:** 1 Internal Medicine, Danbury Hospital, Danbury, USA; 2 Infectious Diseases, Danbury Hospital, Danbury, USA; 3 General Medicine, Universidad Iberoamericana (UNIBE), Santo Domingo, DOM

**Keywords:** cryptococcus neoformans (c. neoformans), central nervous system infections (cns), clinical mycology, immuno-competent, cryptococcus meningitis

## Abstract

Cryptococcal meningitis is a severe fungal infection that primarily affects individuals with compromised immune systems, such as those with the human immunodeficiency virus (HIV) or those undergoing immunosuppressive therapies after organ transplantation. In rare cases, immunocompetent individuals may also be affected by this life-threatening condition.

We present the case of a 64-year-old male patient with no known underlying immune deficiency diagnosed with cryptococcal meningitis, who presented with persistent headaches and subjective fevers. Due to the absence of apparent immunosuppressive conditions or identifiable risk factors during evaluation, our suspicion for fungal meningitis was low. However, the diagnosis was confirmed through CSF fluid analysis, leading to the immediate initiation of guideline-directed treatment with amphotericin and fluconazole.

This case highlights the importance of considering cryptococcal meningitis in the differential diagnosis of persistent headaches, even in patients without known immune compromise. Early recognition and appropriate management are essential to preventing complications and delays in management and guaranteeing optimal outcomes for all our patients.

## Introduction

Cryptococcal meningitis is a fungal infection that primarily affects immunosuppressed patients and is the most common cause of meningitis in individuals with acquired immune deficiency syndrome (AIDS). Symptoms may include typical meningeal signs like headache and neck stiffness, as well as systemic manifestations of disseminated disease such as pneumonia and dermatological findings. Diagnosis is typically confirmed by performing a lumbar puncture, which often reveals elevated opening pressures and the detection of organisms by India ink staining or cerebrospinal fluid (CSF) cryptococcal latex antigen.

While cryptococcal infection is commonly associated with immunosuppression, there have been reported cases of cryptococcal meningitis in immunocompetent patients, and the number of such cases has been increasing in recent years. In the past 20 years, mortality from this disease has reached approximately 600,000 deaths per year. Today, despite diagnostic and therapeutic advances, acute cryptococcal infections of the central nervous system (CNS) continue to carry a significant mortality rate, affecting hundreds of thousands of people each year, irrespective of their access to diagnostic tools or treatment [1].

## Case presentation

A 64-year-old man with a medical history of degenerative lumbar joint disease and benign prostatic hyperplasia (BPH) presented to the emergency department (ED) with a subacute headache, lethargy, and generalized weakness. 

Surgical and social histories were non-contributory, with no engagement in high-risk sexual behavior or IV drug use. In the past month, he traveled to British Columbia, Canada, on a backcountry ski trip along with eight other people. They lodged in a primitive log shack that required a wood-fire stove for heating but otherwise denied exposure to insects, bats, or visits to caves. His symptoms started a week after his return to the US. 

Five days before admission, he presented to his primary care provider with progressive headaches, subjective fevers, and diaphoresis for seven days, which had not improved with over-the-counter analgesics. At that time, his vital signs and physical exam were unremarkable; a complete blood count (CBC), a complete metabolic panel (CMP), a tick-borne disease antibody panel, and blood cultures were obtained. Results were available the next day without electrolyte abnormalities or evidence of infection.

He presented two days later to the ED with fevers, nocturnal sweats, and a frontal headache. On admission, his temperature was 36.8°C, his heart rate was 61 bpm, and his blood pressure was 119/82 mmHg. A thorough physical exam was unremarkable, including a neurologic exam with no presentation of focal deficits, meningismus, photophobia, or aphasia. Blood work results from admission are summarized in Table 1.

**Table 1:** Bloodwork Results of Admission Day BUN = Blood Urea Nitrogen; WBC = White Blood Cells

Variable	Result	Reference Value	Unit
Sodium	134	135 to 145	mmol/L
Potassium	3.9	3.5 to 5.3	mmol/L
Chloride	95	97 to 107	mmol/L
Bicarbonate (HCO3)	28	22 to 29	mmol/L
BUN	19	6 to 23	mg/dL
Creatinine	0.85	0.67 to 1.23	mg/dL
Lactic Acid	2.0	>1.9	mmol/L
WBC	7.5	3.5 to 10.0	x 10^9/L
Hemoglobin	15.7	13.5 to 17.0	g/dL
Hematocrit	44.3	38.0 to 50.0	%
Platelets	191	150 to 400	x 10^9/L
Sedimentation Rate	8	0 to 19	mm/hr

A lumbar puncture and CSF fluid analysis showed lymphocyte-predominant pleocytosis, raising suspicion for a viral central nervous system (CNS) infection (Table 2). Opening CSF pressures were not obtained. An MRI of the brain showed no abnormal parenchymal, meningeal, or ependymal enhancement. He was empirically started on acyclovir and ceftriaxone pending preliminary CSF cultures. On hospital day #3, the viral panel for CSF fluid was negative, and cultures started to grow rare *cryptococcus*.

**Table 2:** Analysis of CSF Fluid Ag = Antigen; CSF = Cerebrospinal Fluid; N/A = Not Applicable; RBC = Red Blood Cells; WBC = White Blood Cells *Sent to a reference lab for further identification, the final result 16 days later showed a rare *Cryptococcus neoformans* complex ** Antigen result available on hospital day #3

Variable	Result	Reference Value
CSF Appearance	Clear	N/A
CSF Color	Colorless	N/A
CSF WBC	11 cells/mm^3^ (H)	0-5 cells/mm^3^
CSF RBC	68 cells/mm^3^	0
CSF PMN	9%	0 to 5 cells/mm^3^
CSF Lymphocytes	82%	0 to 5 cells/mm^3^
CSF Monocytes	9%	0 to 5 cells/mm^3^
CSF Glucose	54 mg/dL	40 to 70 mg/dL
CSF Protein	91 mg/dL	15 to 45 mg/dL
CSF Culture with Gram Stain	Rare Probable *Cryptococcus* Species*	N/A
Crypto Ag CSF	1:1 **	N/A

Further work-up was ordered to assess immune status pending final culture results to determine *cryptococcus* species, including immunoglobulin levels (IgA, IgG, and IgM), lactate dehydrogenase, prostate-specific antigen levels, and HIV antigen/antibody levels, which all yielded unremarkable results. Induction therapy with amphotericin at 3 mg/kg and flucytosine at 25 mg/kg four times a day for two weeks was started.

Unfortunately, flucytosine was not continued after discharge due to cost restrictions, and he was prescribed a fluconazole regimen of 800 mg daily for a total of eight weeks. He was discharged after an uncomplicated 12-day hospital stay with partial resolution of headaches but overall significant clinical improvement, with daily appointments at the infusion center. In outpatient follow-up with the infectious diseases services, the patient is back to baseline clinical status and continues on maintenance dosing of fluconazole (400mg/d) in anticipation of completing a six-month course.

## Discussion

*Cryptococcosis* is defined as the clinical manifestations caused by *Cryptococcus*, an invasive encapsulated yeast that commonly affects immunosuppressed individuals. The most common pathogenic species in humans are *Cryptococcus neoformans* and *Cryptococcus gatti* [1].

Worldwide, about 1 million cases are reported every year, with over 60% mortality. *C. neoformans* usually affects immunocompromised patients due to the unregulated hematogenous spread of *cryptococcal* cells through the blood-brain barrier, presenting clinically as meningoencephalitis, whereas *C. gatti* harbors a stronger association with immunocompetency. Hosts with a regular immune response can eliminate most of the inhaled yeast. *C. gatti*, on the other hand, has a considerably stronger association with pulmonary disease [2].

The most common method of transmission is inhalation of spores through soil, decaying wood, or bird droppings [2], none of which our patient was knowingly exposed to. 

Cryptococcal infection as a cause of meningoencephalitis should be considered in patients with some degree of immune dysfunction, including HIV, malignancy, transplants, autoimmune conditions, diabetes mellitus, alcoholism, or medications [3]. The diagnosis is reached through a lumbar puncture with a CSF profile of low white blood cell counts (WBC) and mononuclear predominance, elevated protein levels, and low-to-normal glucose. Confirmatory testing includes cryptococcal culture and cryptococcal antigen (CrAg) in the spinal fluid. 

According to the Infectious Disease Society of America (IDSA), underlying disease, site of infection, host immunity, and anti-fungal drug toxicity are some of the key factors determining successful management of *cryptococcosis*. Patients are divided into three categories: HIV-infected individuals, organ transplant recipients, and non-HIV-infected/non-transplant hosts. 

IDSA therapy guidelines for cryptococcal meningoencephalitis include induction, consolidation, maintenance, and sometimes suppressive therapy. Mainstay agents include amphotericin B (deoxycholate [AmBd] or lyposomal complex [ABLC]), flucytosine, and fluconazole. Our patient’s symptoms of headache and fever are non-specific but common in cryptococcal meningoencephalitis in immunocompetent individuals [4,5]. For hosts without underlying immune compromise or transplant history, treatment may last from 6-12 months for all stages, as described in Table 3.

**Table 3:** Treatment of Cryptococcal Meningoencephalitis in Non-HIV-Infected and Non-Transplant Hosts AmBD = amphotericin B (deoxycholate; LFAmB = Lipid formulation of AmBd (liposomal AmB and AmB lipid complex). QID = four times a day; CSF: Cerebrospinal fluid; BID = twice a day **AmBd may be substituted for LFAmB in the last two weeks if (+) toxicity ***AmBd may be substituted for LFAmB in the last four weeks if (+) toxicity If persistent, restart the induction phase for 4-10 weeks and increase the dose of AmBd up to 1 mg/kg daily or 6 mg/kg of liposomal amphotericin

Phase	Course	
Induction	AmBd 0.7-1.0 mg/kg daily plus Flucytosine 100 mg/kg QID	Four weeks if: (1) no neurologic complications and (2) CSF yeast cultures are negative after two weeks of treatment** six weeks if (+) neurologic complications***
Consolidation	Fluconazole 400-800 mg daily	Eight weeks
Maintenance	Fluconazole 200 mg [3 mg/kg] daily	Daily for 6-12 months
Relapse	Restart induction phase regimen and add: (1) Fluconazole 800-1200 mg daily or (2) Voriconazole 200-400 mg BID or (3) Posaconazole 200 mg QID or 400 mg BID	10-12 weeks

## Conclusions

The prevalence rates of *Cryptococcus neoformans* in the United States are not consistently reported and may vary depending on geographic location, population, and risk factors. However, it is estimated that there are approximately 7,000 to 10,000 cases of cryptococcal meningitis annually in the United States, with most cases occurring in immunocompromised individuals, such as those with HIV/AIDS or receiving immunosuppressive therapy. Early diagnosis and treatment may help reduce cryptococcal meningitis-related mortality. One way to diagnose cryptococcal infection early in the course of disease is through the detection of serum cryptococcal antigen (CrAg), which can be detected at least three weeks before the onset of neurologic symptoms. 

## References

[1] Maziarz EK, Perfect JR (2016). Cryptococcosis. Infect Dis Clin North Am.

[2] Kwon-Chung KJ, Fraser JA, Doering TL, Wang Z, Janbon G, Idnurm A, Bahn YS (2014). Cryptococcus neoformans and Cryptococcus gattii, the etiologic agents of cryptococcosis. Cold Spring Harb Perspect Med.

[3] Poley M, Koubek R, Walsh L, McGillen B (2019). Cryptococcal meningitis in an apparent immunocompetent patient. J Investig Med High Impact Case Rep.

[4] Perfect JR, Dismukes WE, Dromer F (2010). Clinical practice guidelines for the management of cryptococcal disease: 2010 update by the infectious diseases society of america. Clin Infect Dis.

[5] (2023). Centers for Disease Control and Prevention: C. neoformans Infection Statistics. https://www.cdc.gov/fungal/diseases/cryptococcosis-neoformans/statistics.html.

